# Penile Fracture: A Case Report

**DOI:** 10.31729/jnma.7876

**Published:** 2022-10-31

**Authors:** Prajwala Yogi, Shova Sapkota, Samir Shiwakoti, Udaya Man Singh Dongol, Prajwal Paudyal, Anup Karki

**Affiliations:** 1 Kathmandu Medical College and Teaching Hospital, Sinamangal, Kathmandu, Nepal; 2 Department of Urosurgery, Kathmandu Medical College and Teaching Hospital, Sinamangal, Kathmandu, Nepal

**Keywords:** *masturbation*, *penis*, *sexual intercourse*

## Abstract

Penile fracture is a rare condition with an incidence of 1 case per 175000 United States male population. It can be caused by vigorous sexual intercourse and masturbation. Patients usually present with pain and swelling of the penis and can be diagnosed clinically. It mostly occur as a result of rupture of tunica albuginea of corpora cavernosa. Ultrasound is the most reliable investigation to detect penile fractures. Patients need prompt treatment with exploration and repair of defects to prevent longterm sequelae. Here we present a case of 44 years male who developed a penile fracture following sexual intercourse and underwent surgical exploration and repair.

## INTRODUCTION

Fracture of the penis is one of the rare presentations in urology, accounting for 1 case per 175000 US male population.^1^ It occurs mostly as a rupture of tunica albuginea of corpora cavernosa.^2^ Most common causes of penile fracture include vigorous sexual intercourse and masturbation.^3^ Patient classically presents with eggplant sign which is generalised swelling and ecchymosis of the penis due to subcutaneous hematoma.^4^ Diagnosis of the patient is based on clinical evaluation and should undergo surgical intervention without delay.^5^ Delay in seeking care because of fear and embarrassment may result in the delayed surgical repair which predisposes to adverse sequelae like erectile dysfunction and penile curvature.^6^ Fear and embarrassment can also be the cause of underreporting of penile fracture in developing countries. We hereby present a case of penile fracture presented to the Department of Urosurgery of our hospital.

## CASE REPORT

We present a case of a 44-year male, who presented to Urosurgery outpatient department with a chief complaint of pain in the penis for one day. The pain was acute in onset and started after sexual intercourse. On physical examination, his penis was swollen and deformed. There was no urethral bleeding nor voiding diffiulties suggesting an uncompromised urethra. Then the patient was admitted to the urosurgery ward with the clinical diagnosis of penile fracture and urinary catheterisation was done with foley's catheter of 16 French (F). Further ultrasound (USG) of the penis was done to confirm the diagnosis, which showed defect measuring 2 mm in anterolateral aspect of the left corpus cavernosum at about 4 cm distal from the base at the distal penile shaft region.

**Figure 1:**
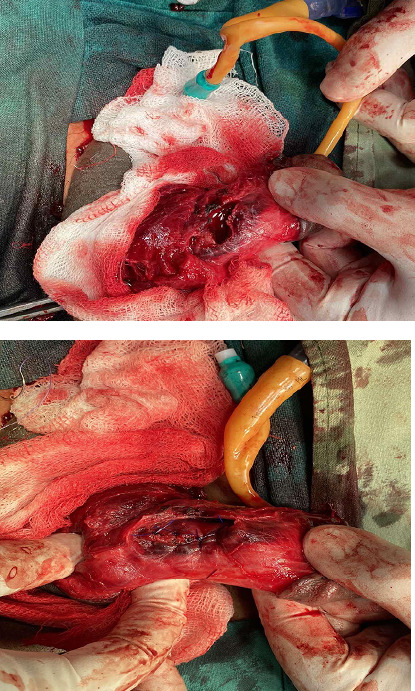
A defect of 2.6 mm in the anterolateral aspect of the left corpus cavernosa with hematoma.

There was an associated focal heterogeneous collection noted surrounding the site of tunica. Diffuse edematous thickening of the penile shaft was present. Proximal and distal shaft of penis showed normal corpus cavernosum and corpus spongiosum appeared normal without defect. He underwent further preoperative investigations like complete blood count, renal function test, urine routine microscopic examination, serology, report of which came normal. On exploration at the operation theatre, a defect of 2.6 mm in the anterolateral aspect of the left corpus cavernosa with hematoma was detected and was repaired with prolene suture (Figure 1).

The patient was prescribed cefixime 200 mg twice a day for 7 days and metronidazole 400 mg thrice a day for 1 week to prevent infection and was advised to take diazepam 5 mg if needed in case of painful erection and then discharged. The patient followed up after 1 week at the outpatient department, on examination the wound was healthy with no discharge, removal of Foley catheter was tried but couldn't be removed. The dressing was done and the patient was advised to follow up after 3 days for Foley's catheter removal.

On follow-up dressing was done, and after wound healing suture was removed.

## DISCUSSION

Penile fracture is a disruption or tear of the tunica albuginea of the corpus cavernosum in the penis, occurring in isolation or with urethral tear.^2^ It is an uncommon presentation to Urology departments with incidence of 1 in 175,000 in US male population, However, it is still underreported in developing countries.^1^

Most commonly, it occurs during sexual intercourse when the penis slips out of the vagina and strikes the perineum or against the pubic bone, which causes sudden bending of the penis resulting in a buckling injury to the penis and a tearing of the tunica albuginea of the corpus cavernosum.^7^ Most common site of rupture occurs on the lateral side of the proximal corpora, but it can occur anywhere along the corpora and produce a variety of swelling patterns.^8^ In our case, the defect was in anterolateral aspect of the left corpus cavernosum at about 4 cm distal from the base at the distal penile shaft region. Other causes include rolling over in bed on to the erect penis, forced flexion to achieve detumescence and external blunt trauma, anal intercourse and masturbation.^9^ The tunica albuginea can resist rupture until intra-cavernous pressure increases to more than 1500 mm Hg due to its marked tensile strength. The thickness of tunica albuginea decreases from 2 mm, in a flaccid penis to 0.25 mm, in erect penis and sudden increase in intracorporeal pressure due to trauma during an erection could easily result in rupture.^10^

Penile fracture can be diagnosed on clinical assessment alone. The patient reports a "popping" sound after buckling of the penile shaft followed by pain, rapid detumescence, and subcutaneous hematoma leading to an "eggplant deformity".^11^ In equivocal cases, ultrasonography is the preferred imaging of choice which was also done in our case. It is readily available, noninvasive, inexpensive, and accurate. Penile ultrasound is most useful for ruling out fracture in patients with low clinical suspicion. Other modes of investigation employed in the case of penile fracture are MRI, cavernosography, urethrography and flexible cystoscopy.^12^ Retrograde urethrography should be performed if urethral injury is suspected based on the presence of blood at the meatus, hematuria of any form, dysuria, or urinary retention.^13^

Fracture of penis is a rare urological emergency which requires prompt surgical exploration and repair which includes evacuation of the hematoma, identification of the tunica injury, local corpora debridement, closure of the tunica lacerations, and ligation of any disrupted vasculature.^8^ For partial urethral tears,urethral catheterization, primary closure with nonabsorbable suture, or suprapubic cystostomy tube can be done. Immediate surgery reduces long-term complications; post-traumatic penile curvature remains the most common long-term complaint.^3^
